# Supplementary material for: Two Clubroot-Resistance Genes, Rcr3 and Rcr9wa, Mapped in Brassica rapa Using Bulk Segregant RNA Sequencing
Source: Int J Mol Sci. 2020 Jul 16;21(14):5033. doi: 10.3390/ijms21145033 (PMC7404267; doi:10.3390/ijms21145033)

Supplementary Figure S1. Allelic discrimination plots (KASP method) of BC1 populations (dark blue and green) analyzed using KASP SNP marker; ACDC (susceptible, light blue) and 96-6990-2 (resistant, red) are homozygous parental lines.

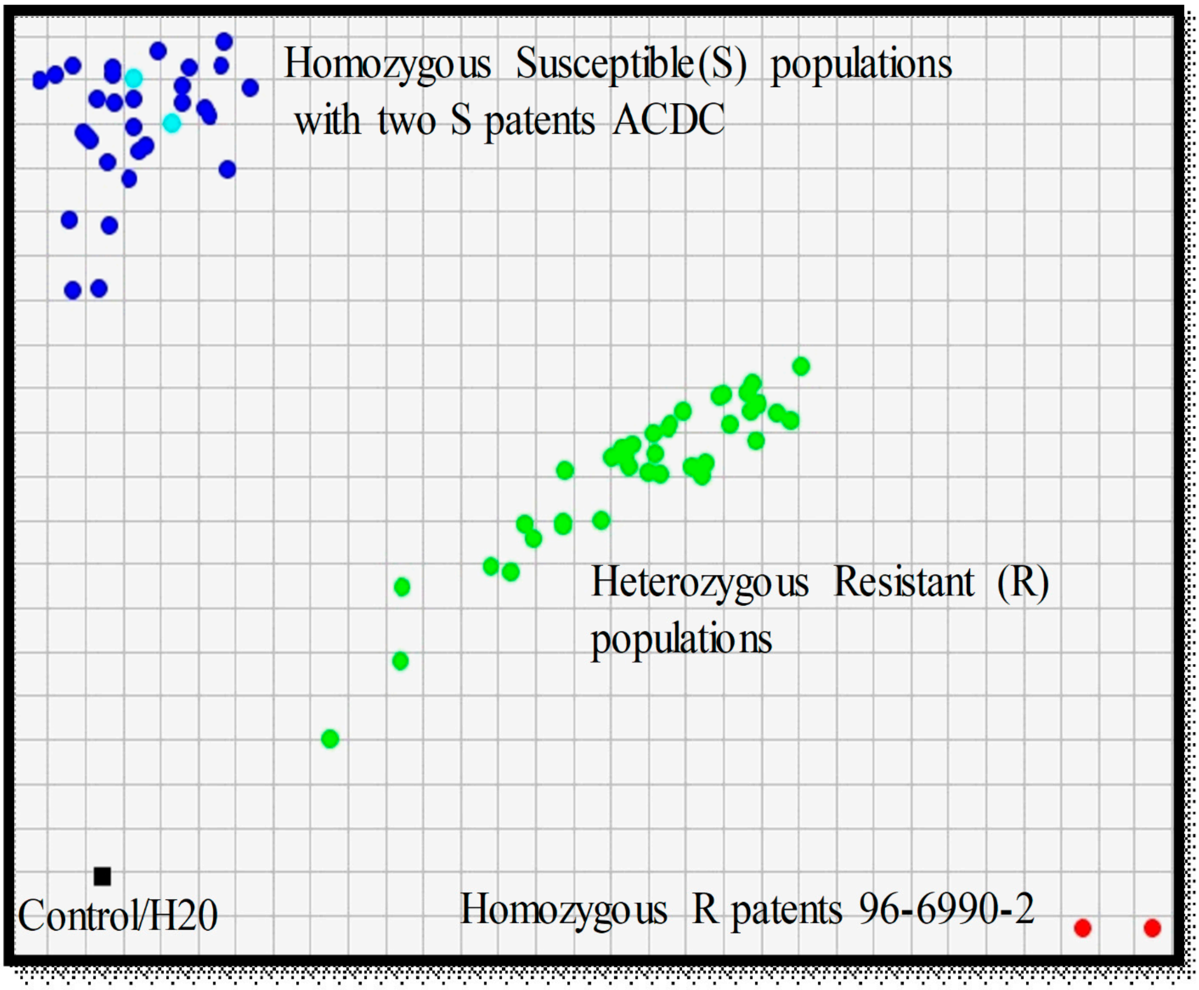

Supplementary Figure S2. Cluster dendrogram showing genetic distance of selected Brassica genotypes.

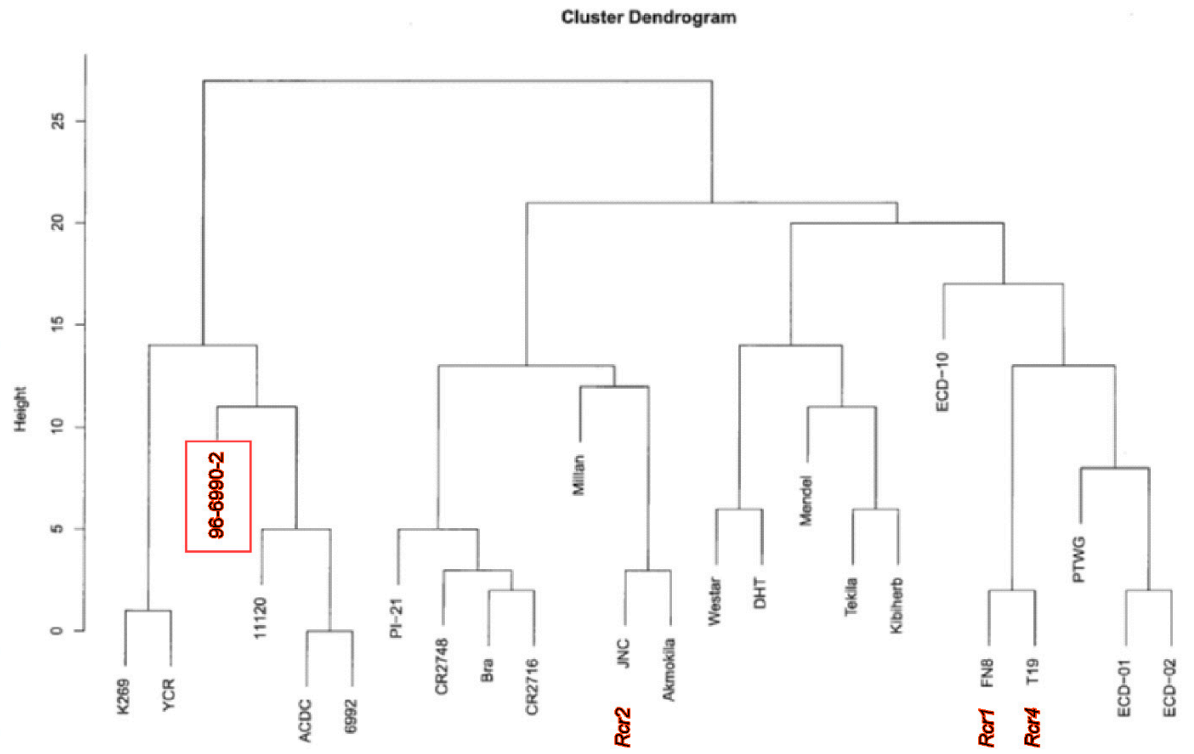

Supplement: Supplementary file 1 [file ijms-21-05033-s001.zip › Karim et al. 2020-June 22_Supp Figure 1-2.pdf]
